# Rare Invasive Yeast Infections in Greek Neonates and Children, a Retrospective 12-Year Study

**DOI:** 10.3390/jof6040194

**Published:** 2020-09-28

**Authors:** Maria Noni, Angeliki Stathi, Aristea Velegraki, Mika Malamati, Alexandra Kalampaliki, Levantia Zachariadou, Athanasios Michos

**Affiliations:** 1 First Department of Pediatrics, Division of Infectious Diseases, “Aghia Sophia” Children’s Hospital, National and Kapodistrian University of Athens, 11527 Athens, Greece; marianoni21@gmail.com; 2 Department of Microbiology, “Aghia Sophia” Children’s Hospital, 11527 Athens, Greece; angelikiestathi@gmail.com (A.S.); matina_60@yahoo.gr (M.M.); alexkal2014@hotmail.com (A.K.); levantia@otenet.gr (L.Z.); 3 Mycology Research Laboratory and UOA/HCPF Culture Collection, Microbiology Department, Medical School, National and Kapodistrian University of Athens, 11527 Athens, Greece; aveleg@med.uoa.gr; 4 Mycology Laboratory, Biomedicine S.A., 11526 Athens, Greece

**Keywords:** fungi, invasive, children, rare

## Abstract

Although *Candida* species remain the leading cause of invasive fungal infections (IFI), the list of other isolated fungal pathogens is increasing. The aim of the study was to report cases of IFI caused by rare yeasts in the largest tertiary Greek pediatric hospital. A retrospective study was performed from 6/2008–6/2020 regarding IFI caused by rare species. Identification of isolates was attained by conventional, molecular, and MALDI TOF MS methods, and susceptibility testing was performed according to the Clinical and Laboratory Standards (CLSI) methodology. During a 12-year period, 14 different rare fungal species in 33 neonates and children with IFI hospitalized in intensive care and oncology units were isolated from blood, central catheters, peritoneal, pleural, or pericardial fluid specimens. It is the first time for IFI caused by *Wickerhamomyces anomalus (Candida pelliculosa), Pichia fermentans (Candida lambica), Yarrowia (Candida) lipolytica, Pichia (Hansenula) kluyveri, Rhodotorula mucilaginosa, Wickerhamiella (Candida) pararugosa* and *Cyberlindnera (Candida) fabianii* in Greek neonates and children to be reported. For most of these rare fungal species isolated in the present study, no official antifungal breakpoints have been defined, and there are no guidelines for their treatment. Clinical laboratories should be aware of uncommon and emerging yeast pathogens and be able to detect them with molecular and proteomic methods.

## 1. Introduction

Invasive fungal infections (IFI) occur mainly in neonates and children who are immunocompromised or in a critically ill condition [1,2]. These infections are characterized by high rates of morbidity and mortality, and thus, timely diagnosis and initiation of appropriate antifungal therapy is always considered crucial [3].

*Candida* species remain the leading cause of IFI among pediatric population [4]. Several less frequently isolated yeast species have been also implicated in IFI such as species that belong to the yeast genera *Cryprococcus, Rhodotorula, Malassezia, Geotrichum*, etc. [5,6]. Some of them are considered frequent colonizers of skin or mucosal surfaces. Their clinical significance varies from superficial infections in normal hosts to invasive infections in immunocompromised individuals. Because of the rare incidence of IFI, published data mostly consist of case reports and small case series, and thus, management recommendations are derived mainly from clinical experience [6]. Given the complexity of the patients at risk for infection and the increasing array of rare fungal pathogens with intrinsic resistance to antifungal agents, treatment remains challenging.

The aim of the study was to present epidemiological data of IFI caused by uncommon species in the largest tertiary Greek pediatric hospital over a 12-year period.

## 2. Materials and Methods

### 2.1. Study Design

A retrospective cohort study of IFI caused by rare fungal species was performed from June 2008 to June 2020 at “Aghia Sophia” Children’s Hospital in Athens. This is a 750-bed tertiary hospital that is a referral center for almost 75% of the Greek pediatric population and includes 3 neonatal intensive care units (NICU), 1 pediatric ICU (PICU), 2 hematology–oncology units (HOU), 1 bone marrow transplantation unit, 5 surgical units (SU), and 1 department of cardiovascular surgery (DCS). All records from the Department of Microbiology were reviewed as well as patients’ medical records when available. All the non-*Candida* yeasts were defined as rare fungal species.

IFI was defined according to the European Organization for Research and Treatment of Cancer/Invasive Fungal Infections Cooperative Group and the National Institute of Allergy and Infectious Diseases Mycoses Study Group (EORTC/MSG) Consensus Group consensus revised definitions [7]. Proven fungal infection was defined as the isolation of fungi in blood, central catheters, peritoneal, pleural, or pericardial fluid specimens. Date of infection was defined as the date of clinical disease connected to the first positive culture for fungi that met disease criteria [7]. If multiple episodes of invasive fungal infection occurred in a single patient, episodes separated by clinical and microbiological resolution (defined by ≥14 days) were treated as a new episode.

### 2.2. Mycology and Antifungal Susceptibility Test

Cerebrospinal fluid (CSF) and Bronchoalveolar Lavage (BAL) were inoculated directly on Sabouraud Dextrose Agar (SDA) (Bioprepare, Keratea, Greece) and in Sabouraud Dextrose Broth (SDB) (prepared in house), whereas blood, peritoneal, and pleural fluid incubation in the BD BACTEC FX^TM^system (Becton Dickinson, Franklin Lakes, NJ, USA). Yeast pathogens from positive cultures were sub-cultured onto SDA, Candida Chrom Agar (CCA) (Bioprepare), and on selective mDixon agar (prepared in house), for Malassezia isolates, processed for identification by micromorphology and carbohydrate assimilation testing with API^®^32 C, (BioMérieux, Craponne, France), enzymatic RapID^TM^YeastPlus System (ThermoScientific^TM–^Remel, Lenexa, KS, USA), and Vitek2 (BioMerieux).

Identification of non-identified and confirmation of identified isolates was done by matrix-assisted laser desorption/ionization time-of-flight MALDI-TOF MS (Bruker, Karlsruhe, Germany) and where appropriate by sequencing of the ITS region, D1/D2 domain of the Large Subunit (LSU) rDNA gene, while *Trichosporon* species were genotyped by sequencing the Intergenic Spacer 1 (IGS) region and the D1/D2 domain [8]. Following identification, the pure isolates were frozen at −70 °C.

For susceptibility testing, isolates were freshly sub-cultured and analyzed. Minimal inhibitory concentrations (MICs) values were measured for amphotericin B, flucytosine, fluconazole, itraconazole, posaconazole, voriconazole, and caspofungin by broth microdilution method Micronaut-AM (Merlin Diagnostic, Bornheim, Germany) according to the manufacturers’ instructions and also by gradient concentration MIC method of the antifungal drug in strips (Liofilchem, Roseto degli Abruzzi, Italy), and for some cases, E-test (BioMérieux), applied on inoculated 0.5 McFarland yeast suspension turbidity 90 mm diameter RPMI agar plates with MOPS in a 1.5% agar base, supplemented with 2% glucose (Liofilchem,) and incubation at 35 °C for 24–48 h. Susceptibility to isavuconazole was determined by strips only. MICs were read upon the intersection point of the growth with concentration scale, taking into consideration the “trailing” effect where appropriate. Regarding *Malassezia* species, MICs were determined only by gradient MIC method on modified RPMI 1640 agar according to previously published protocol [9].

### 2.3. Ethical Approval and Informed Consent

The study was reviewed and approved (5878/07-03-2014) by The Hospital’s Research Ethics Board.

## 3. Results

During the study period, 49 incidences of rare fungi species were detected in 45 neonates and children with IFI and non-IFI infections. Fourteen different rare fungal species—cases of filamentous fungi have not been included—were isolated in 33 out of 416 neonates and children (7.9%) with IFI hospitalized in oncology and intensive care units. These fungi were isolated from blood, central catheters, peritoneal, pleural, or pericardial fluid specimens. Yeast species that were isolated for bronchial samples were not included in the analysis. Epidemiological and clinical characteristics of neonates and children with IFI and fungal species isolated are presented in Table 1.

IFI caused by *Trichosporon* species were diagnosed in eight children. *Trichosporon asahii* was isolated in in six and *Trichosporon mucoides* in two patients. Two patients were hospitalized in the Bone marrow transplant unit (BMTU), two in HOU, one in SU, one in DCS, one in PICU, and one in NICU. *T. mucoides* exhibited lower MIC values for amphotericin B than *T. asahii* (Table 2). Both species showed increased MIC values for echinocandins and flucytosine. Regarding azoles, most *Trichosporon* species showed low MIC values, except for fluconazole.

*Saccharomyces cerevisiae (Candida robusta)* caused IFI in six patients. Two patients were hospitalized in the NICU, two in the PICU, one in the DCS, one in the HOU, and all of them were females. All isolates were found susceptible to amphotericin B, echinocandins, voriconazole, isavuconazole, and flucytosine (Table 2). Isolates exhibited a posaconazole MIC value from 1.0 to 6.0 mg/dL, while increased MIC for fluconazole were detected in most cases. Repetitions were performed with posaconazole and constant differences were observed both with strips and microdilution method. Thus, it was hypothesized that the gradient MIC method with strips may not be the most appropriate, mainly in terms of *S. cerevisiae* growth and posaconazole activity in media. Nevertheless, despite the differences, the MICs remain low and comparable to those in other studies. Since the microdilution is the reference method, and in absence of clinical breakpoint, all but one isolates considered sensitive to posaconazole [10,11].

Among the six IFI cases caused by *Cryptococcus* and former *Cryptococcus* species that were detected, the responsible isolated species were *Naganishia albida* (*n* = 3), *Cryptococcus uniguttulatus* (*n* = 2), and *Cryptococcus terreus* (*n* = 1). *Cryptococcus* species were isolated from blood cultures of three patients and *N. albida* from two blood cultures and a pleural fluid sample of one patient with congenital heart disease. Three patients were hospitalized in the HOU, one in the DCS, one in the NICU, and one in the BMTU. All of them were males. Isolates exhibited an amphotericin B MIC value from 0.38 to 1.5 mg/L. Flucytosine, fluconazole, and as expected, echinocandins showed limited in vitro activity against *N. albida*, *C. uniguttulatus*, and *C. terreus* (Table 2).

Regarding *Malassezia furfur* isolates*,* they were detected in blood cultures and one central catheter of five patients. Three of them were hospitalized in the NICU, one patient in the HOU, and one in the BMTU. The lowest MIC values were found for amphotericin B, itraconazole, posaconazole, and isavuconazole (Table 2). Interestingly, all isolates were found resistant to echinocandins and flucytosine, while three isolates exhibited increased MIC values for fluconazole.

*Wickerhamomyces anomalus (Candida pelliculosa*) caused IFI in two patients who were hospitalized in the HOU and in the DCS. Both strains showed low MIC values to many antifungal agents including itraconazole, voriconazole, posaconazole, echinocandins, and flucytosine (Table 2).

*Pichia fermentans (Candida lambica)* and *Yarrowia (Candida) lipolytica* were isolated from patients’ samples who were hospitalized in ICU. Both species exhibited low MIC values against the majority of antifungal agents, except fluconazole, which showed limited in vitro activity against *Yarrowia (Candida) lipolytica*. *Pichia (Hansenula) kluyveri*, *Rhodotorula mucilaginosa*, and *Wickerhamiella* (*Candida) pararugosa* were isolated from patients’ blood samples with history of malignancy. *P. kluyveri* and *W. pararugosa* exhibited low MIC values for all antifungals except fluconazole. Regarding *R. mucilaginosa*, the fungus was susceptible to amphotericin B, flucytosine, and isavuconazole. Finally, *Cyberlindnera (Candida) fabianii* was isolated from a neonate who was hospitalized in the NICU for a long period due to pulmonary dysplasia and the fungus was isolated in blood samples as well as in bronchial secretions and in rectal carriage. The fungus exhibited low MIC values to all antifungal agents (Table 2).

## 4. Discussion

Identification of rare and emerging causes of fungaemia and disseminated infections in neonates and children, during an extended 12-year period in the largest tertiary Pediatric Hospital of Greece, is presented in the present study. This is the first report of IFI in Greek neonates and children caused by very rare species such as *W. anomalus*, *P. fermentans, Y. lipolytica*, *P. kluyveri*, *R. mucilaginosa*, *W. pararugosa* and *C. fabianii*. The study provides useful antifungal susceptibility data that may guide selection of appropriate antifungal therapy in the future.

*Trichosporon* species can colonize many different systems of the human body including the skin, gastrointestinal system, and respiratory system and are capable of causing both superficial and invasive infections [12]. Regarding children population, invasive disease is predominantly found in patients with hematologic disorders as well as in premature neonates with *T. asahii* being the leading cause [13]. Azoles are considered as the primary choice among antifungal agents for the treatment of invasive trichosporonosis, while several species, including *T. asahii*, are resistant in vitro to amphotericin B, flucytosine, and echinocandins [6]. As expected, isolates exhibited increased echinocandin MIC values.

*S. cerevisiae* can be found as a harmless and asymptomatic colonizer of mucosal surfaces in normal individuals. However, it might also be responsible for IFI particularly in vulnerable patients receiving probiotics or critically ill patients with severe underlying diseases such as cancer [14]. Few cases in the literature refer to pediatric population, and all were treated effectively [15,16,17]. The antifungal agent of choice has not been established. *S. cerevisiae* is usually susceptible to amphotericin B and flucytosine, while MICs for fluconazole and itraconazole present a wide range [6]. Regarding our detected isolates, all were susceptible to amphotericin B and flucytosine and exhibited increased MIC values for fluconazole. The clinical experience with echinocandins is limited, however there have been reports from adults with successful treatment based on these agents [17,18].

The genus *Cryptococcus* comprises approximately 70 species, which are able to cause infections in humans as well as in animals [6]. *Cryptococcus neoformans* and *Cryptococcus gatti* are considered the major pathogens. However, other *Cryptococcus* species (e.g., *C. uniguttulatus*) and *N. albida*, former *Cryptococcus albidus,* are prevalent worldwide and have emerged as opportunistic pathogens over the last few years [19]. Epidemiological studies suggest that patients with impaired cell-mediated immunity or invasive devices are predisposed to these infections [20]. Non-*neoformans* cryptococci have been reported to cause infection in many organ systems, particularly in bloodstream and central nervous system. The choices and duration of treatment for non-*neoformans* cryptococci infections depend on the anatomical sites of involvement, the host-immune status, response to therapy, and the severity of infection [21]. In general, amphotericin B is a mainstay of the treatment, while echinocandins are inactive against *Cryptococcus* species [6]. Azole agents are reasonable alternatives for patients with less severe infection. Interestingly, our cases were found resistant to fluconazole and flucytosine. According to the literature, MICs values of flucytosine, fluconazole, and other azoles are elevated in many reports [22,23,24]. Regarding fluconazole, resistance is more common in patients who had already received azole prophylaxis [20,25]. Although this might indicate reduced susceptibility, it is not known if this observation correlates with a worse outcome [26]. In view of the different susceptibility profiles of *Cryptococcus* species, characterization at species level of clinical isolates and detailed drug susceptibility testing are considered compulsory.

The genus *Malassezia* is classified into at least 17 species. *M. furfur* is a frequent colonizer of human skin, which can cause various types of skin infections including folliculitis and seborrheic dermatitis. It may, also, lead to severe systemic conditions such as catheter-related fungaemia [27]. Colonization of the skin and subsequent extension to central venous catheters seems to happen more often in neonates than adults. Among the most important risk factors are exposure to lipid-rich intravenous infusions, low birth weight, and prolonged duration of antimicrobial exposure [28]. Systemic infections may, also, occur in patients with various forms of immunosuppression and underlying diseases such as hematological malignancies [29]. Therapeutic management is based mainly on amphotericin B and fluconazole. In vitro resistance to flucytosine and echinocandins seems to be a common finding and was detected in our isolates as well [6,30,31].

*W. anomalus* is an opportunistic pathogen that rarely causes fungaemia. Although many cases have been reported as sporadic, epidemics have also been detected in pediatric intensive care units [32,33,34]. As there is still no consensus on breakpoints for antifungal agents, amphotericin B seems optimal for such infections as most of reported cases have shown good sensitivity to this agent [34,35]. Regarding azoles, many published studies have reported low in vitro susceptibilities to itraconazole, fluconazole, and voriconazole [35,36]. However, Barchiesi et al. reported that 46 strains isolated in 37 patients had low sensitivity to azoles [37].

*R. mucilaginosa* is the most common species of the genus *Rhodotorula*, which consist of 46 species. It is widely distributed in the environment and is a constituent of the normal respiratory system, gastrointestinal, genital flora, and skin. It has emerged as an important cause of nosocomial and opportunistic infections, particularly in immunocompromised patients [38]. Predisposing factors for *Rhodotorula* infection are the presence of central venous catheter and underlying malignancy [39]. Amphotericin B is the drug of choice for the treatment of serious fungaemia followed by flucytosine. As there are reports of moderate susceptibility to echinocandins and fluconazole, these agents should not be considered as appropriate therapy [40].

*C. fabianii* has been described as a yeast with low virulence [41]. However, recently, few case reports have highlighted its pathogenic activity in high-risk patients, thus recognizing that fungus is an emerging pathogen associated with fungaemia, sepsis, and multiple organ dysfunction syndrome. Interestingly, half of reported cases occurred in pediatric patients, while an outbreak of fungaemia involving preterm neonates has also been reported [42,43]. Although data on antifungal susceptibility of *C. fabianii* are limited, it appears that fluconazole, itraconazole, and posaconazole are less active compared to voriconazole, 5-flucytosine, and echinocandins [44]. However, according to previous reports, the fungus has the ability to develop resistance to different antifungals such as azoles, echinocandins, and amphotericin B [45]. To our knowledge, we report the first case of fungaemia caused by *C. fabianii* in Greek children.

*Y. lipolytica* is an ascomycetous yeast found ubiquitously in the environment and meat products [46]. Although it was considered to be of low virulence, it has been also recognized to cause nosocomial clusters of infections [47]. The fungus has the ability to adhere and colonize CVC lines through slime production leading subsequently to candidemia. The major knowledge gap is the optimal management strategy [46]. Although the accepted guidelines for candidiasis recommend catheter removal and intensive treatment with antifungal agents, some clinicians suggest that catheter removal alone might be sufficient for *Y. lipolytica* catheter-related infection [47,48,49].

*P. fermentans* has rarely been implicated in invasive infections and only few cases have been published up to now [50,51]. Regarding our case, the fungus was detected in a blood sample of an infant who was hospitalized in the ICU and was susceptible to many antifungal agents including amphotericin B, flucytosine, and voriconazole, which is in line with the already published data [52].

*W. pararugosa* are uncommon species, which have not been described in detail in the literature. Until today, only four cases have been reported with bloodstream infection caused by this fungus [53,54,55]. Two of them were reported in Qatar and were isolated from blood samples of a 6-month-old infant with a history of intrauterine growth restriction and a 5-year-old patient. In our case, the fungus was susceptible to most of antifungal agents, while it exhibited high MIC for fluconazole which had, also, been detected in cases reported from Qatar.

Finally, *P. kluyveri* has been reported recently as the responsible pathogen for causing oral infection in an Iranian patient with hematological malignancy [56]. The fungus showed low MICs to fluconazole, amphotericin B, and anidulafungin and showed increased MIC to caspofungin. In our case, this fungus caused fungaemia in a male child with hematological malignancy. The isolate was found resistant to fluconazole, while it showed low MICs to all the other antifungal agents.

Conventional laboratory techniques usually cannot identify rare fungal species, such as those described in the present study. Use of novel molecular methods such as MALDI-TOF and sequencing of multiple genomic regions are very useful for the identification of uncommon fungal pathogens [57]. Regarding the antifungal susceptibility testing, it was observed that MIC values were approximately the same by broth microdilution method and also by gradient concentration of the antifungal drug in strips except the difference in MIC values of amphotericin B that was detected in one Trichosporon isolation. However, broth microdilution method is considered as the gold standard method.

Limitation of the present study is that it is a retrospective single-center study, although it represents data from the largest Greek tertiary pediatric center. Due to the extended study period, certain clinical parameters and outcomes were not feasible to be documented from the children’s records.

## 5. Conclusions

As IFI are associated with high mortality, it is crucial to include fungi in our differential diagnosis and identify the specific fungal species. The list of emerging fungal pathogens is growing, and thus, both microbiologists and clinicians should be aware epidemiology and possible approaches to therapy.

## Figures and Tables

**Table 1:** Epidemiological and clinical characteristics and yeasts species isolated from neonates and children diagnosed with invasive fungal infections (IFI) in Athens, 6/2008–6/2020.

Case	Month/Year	Yeasts Species	Age	Gender	Unit	Sample	Medical History
1	8/2019	*Saccharomyces cerevisiae* (*Candida robusta*)	2.5 months	female	DCS	blood, central catheter	congenital heart disease, renal failure, surgical history
2	5/2019		14 months	female	PICU	blood	pneumococcal meningitis
3	12/2012		7 years	female	HOU	blood	acute lymphoblastic leukemia
4	4/2010		9 months	female	PICU	blood	myeloblastoma
5	4/2010		less than 12 months	female	NICU	blood	NK
6	3/2010		7 months	female	NICU	blood	jejunal atresia, surgical history
7	10/2017	*Trichosporon asahii*	2.5 years	female	HOU	blood	malignancy of the yolk sac, surgical history
8	3/2015		11 years	male	SU	peritoneal fluid	peritonitis due to appendicitis, surgical history
9	4/2020		1 month	female	NICU	catheter	congenital heart disease, pacemaker insertion, hypotonia, surgical history
10	7/2018		14 years	male	BMTU	blood	acute lymphoblastic leukemia relapse after BMT
11	12/2014		10 years	female	BMTU	pleural fluid	acute lymphoblastic leukemia
12	6/2011		10 years	female	PICU	blood, bronchial secretion, pericardial fluid	Blackfan-Diamond anemia, renal failure
13	12/2009	*Trichosporon mucoides*	2 months	male	DCS	pericardial fluid	congenital heart disease
14	9/2009		10 years	male	HOU	peritoneal fluid	acute lymphoblastic leukemia
15	1/2018	*Naganishia albida*	2 years	male	HOU	blood	acute lymphoblastic leukemia
16	6/2016		5 years	male	BMTU	blood	acute lymphoblastic leukemia
17	1/2016		24 days	male	DCS	pleural fluid	congenital heart disease
18	10/2016	*Cryptococcus uniguttulatus*	15 years	male	HOU	blood	osteosarcoma, surgical history
19	10/2016		13 years	male	HOU	blood	acute lymphoblastic leukemia
20	4/2009	*Cryptococcus terreus*	6 days	male	NICU	blood	necrotizing enterocolitis, surgical history
21	10/2012	*Malassezia furfur*	13 years	male	HOU	blood	acute myeloidleukemia, total parenteral nutrition
22	4/2020		4 years	male	BMTU	central catheter	primary immunodeficiency, VP shunt
23			4 month	male	NICU	blood	prematurity, Hirschsprung disease, colostomy
24	12/2016		3 months	male	NICU	blood	congenital heart disease, duodenal atresia
25	12/2013		12 days	female	NICU	blood	necrotizing enterocolitis, surgical history, total parenteral nutrition
26	7/2013	*Wickerhamomycesanomalus* (*Candida pelliculosa*)	3 years	male	HOU	peritoneal fluid	acute lymphoblastic leukemia
27	2/2019		4 months	female	DCS	blood, central catheter	prematurity, congenital heart disease, respiratory distress syndrome
28	5/2010	*Pichia fermentans* (*Candida lambica*)	less than 12 months	female	PICU	blood	NK
29	6/2008	*Pichia* (*Hansenula*) *kluyveri*	8 years	male	HOU	blood	acute lymphoblastic leukemia
30	1/2020	*Rodotorulamucilaginosa*	12 years	male	BMTU	blood	acute lymphoblastic leukemia
31	1/2018	*Cyberlindnera* (*Candida*) *fabianii*	1 month	male	NICU	blood	pulmonary dysplasia
32	1/2014	*Yarrowia* (*Candida*) *lipolytica*	12 months	female	PICU	pleural fluid	NK
33	10/2008	*Wickerhamiella* (*Candida*) *pararugosa*	3 years	female	HOU	blood	acute lymphoblastic leukemia

BMTU, bone marrow transplant unit; DCS, department of cardiovascular surgery; HOU, hematology and oncology unit; NICU, neonatal intensive care unit; PICU, pediatric intensive care unit; SU, surgical unit; NK, not known; VP, ventriculoperitoneal.

**Table 2:** Antifungal minimal inhibitory concentrations for isolated species.

	Amphotericin B		Fluconazole		Itraconazole		Voriconazole		Posaconazole		Caspofungin		Anidulafungin		Micafungin		Flusytosine		Isavuconazole
	Microdilution	Strips	Microdilution	Strips	Microdilution	Strips	Microdilution	Strips	Microdilution	Strips	Microdilution	Strips	Microdilution	Strips	Microdilution	Strips	Microdilution	Strips	Strips
*Saccharomyces cerevisiae* (*Candida robusta*)	0.125	0.016	2	12	1	4	0.015	0.064	0.125	1.5	0.125	0.125	0.031	0.008	0.0625	0.016	0.0625	0.016	0.094
	0.125	0.125	128	256	2	32	0.25	0.38	4	6	0.125	0.19	0.015	0.064	0.031	0.032	0.0625	0.016	0.019
	-	0.03	-	4	-	2	-	0.12	-	1	-	0.25	-	0.012	-	0.25	-	0.75	0.032
	0.125	0.047	3	12	4	4	-	0.19	0.047	1.5	0.125	0.25	0.125	0.023	0.125	0.023	0.0625	0.047	0.125
	0.125	0.25	2	3	4	4	0.015	0.032	0.25	1.5	0.0625	0.25	0.031	0.016	0.125	0.047	0.0625	0.008	0.023
	0.125	0.5	4	8	4	3	0.031	0.25	0.25	1.5	0.25	0.25	0.125	0.016	0.25	0.016	0.0625	0.016	0.047
*Trichosporonasahii*	0.125	32	4	-	0.03	1.5	0.01	0.064	0.031	0.5	8	32	8	32	8	32	8	32	0.125
	-	32	-	64	-	32	-	1.5	-	32	-	32	-	32	-	32	-	32	1
	0.5	1	4	8	0.06	0.25	0.031	0.094	0.06	0.1	8	32	8	32	8	32	8	32	1
	8	32	6	16	0.031	1.5	0.031	0.125	0.25	0.5	16	32	8	32	8	32	8	32	0.5
	1	1	32	256	0.06	0.047	0.06	0.094	0.125	0.25	4	32	8	32	8	32	8	32	1
	6	8	16	6	4	4	0.19	0.19	0.031	0.5	8	32	8	32	8	32	8	32	0.094
*Trichosporonmucoides*	0.5	0.094	2	1.5	0.031	0.5	0.015	0.064	0.031	0.19	8	8	8	32	8	32	8	32	0.064
	0.5	0.094	16	16	0.5	0.19	0.5	0.064	0.125	0.094	4	32	8	32	8	32	8	32	0.75
*Naganishia albida*	-	0.38	-	256	-	1	-	0.38	-	0.5	-	32	-	32	-	32	-	32	0.38
	-	1.5	-	256	-	0.5	-	0.75	-	0.75	-	32	-	32	-	32	-	32	0.75
	-	1.5	-	256	-	0.38	-	0.19	-	0.25	-	32	-	32	-	32	-	32	1
*Cryptococcus uniguttulatus*	-	1.5	-	256	-	0.5	-	0.75	-	0.5	-	32	-	32	-	32	-	32	0.25
	-	1.5	-	256	-	0.5	-	0.5	-	0.5	-	32	-	32	-	32	-	32	0.5
*Cryptococcus terreus*	0.125	1.5	128	256	0.031	0.5	0.125	0.5	0.25	0.5	8	32	8	32	8	32	8	32	1
*Malassezia furfur*	-	0.19	-	256	-	0.032	-	0.023	-	0.064	-	32	-	32	-	32	-	32	0.125
	-	0.25	-	256	-	0.016	-	32	-	0.012	-	32	-	32	-	32	-	32	0.19
	-	0.5	-	1	-	0.094	-	32	-	0.094	-	32	-	32	-	32	-	32	0.75
	-	0.125	-	64	-	0.125	-	0.064	-	0.064	-	32	-	32	-	32	-	32	0.125
	-	0.19	-	0.25	-	0.125	-	0.094	-	0.094	-	32	-	32	-	32	-	16	0.5
*Wickerhamomycesanomalus* (*Candida pelliculosa*)	-	2	-	4	-	0.094	-	0.5	-	0.19	-	0.38	-	0.125	-	1	-	0.125	0.004
	-	0.094	-	1.5	-	0.064	-	0.047	-	0.125	-	0.125	-	0.004	-	0.008	-	0.064	0.002
*Pichia fermentans* (*Candida lambica*)	0.5	0.047	1	1.5	0.031	0.032	0.008	0.047	0.008	0.064	0.25	0.75	0.5	2	0.25	0.75	0.0625	0.016	0.004
*Pichia* (*Hansenula*) *kluyveri*	-	0.048	-	64	-	0.5	-	0.25	-	0.5	-	0.125	-	0.002	-	0.002	-	1	0.004
*Rodotorulamucilaginosa*	0.25	0.38	128	256	1	8	0.25	32	0.5	8	4	32	8	32	8	32	0.06	0.75	0.5
*Cyberlindnera* (*Candida*) *fabianii*	0.125	0.125	0.25	0.75	0.031	0.19	0.008	0.047	0.008	0.38	0.0625	0.19	0.031	0.094	0.031	0.047	0.0625	0.032	0.004
*Yarrowia* (*Candida*) *lipolytica*	0.25	0.5	16	16	0.031	0.06	0.031	0.03	0.0625	0.06	0.125	0.125	0.125	0.03	0.0625	0.03	0.5	1	0.016
*Wickerhamiella* (*Candida*) *pararugosa*	0.125	0.38	4	4	0.031	0.125	0.015	0.094	0.015	0.125	0.125	0.125	0.125	0.003	0.125	0.006	0.25	0.016	0.125
